# Measurement of heart rate and respiratory rate using remote photoplethysmography in paediatrics: a prospective comparative trial protocol – ‘rMonitoped1’

**DOI:** 10.1136/bmjopen-2025-104840

**Published:** 2026-03-19

**Authors:** Sébastien Haas-Ferrua, Hugo Giaccardi, Barbara Ancey, Emma Freyssinet, Emma Baranton, Aline Joulie, Imad Bendimerad, Marco Olla, Fabrice De Oliveira, Abdelhak Moussaoui, Laurent Boyer, Eric Fontas, Antoine Tran

**Affiliations:** 1 Paediatric Emergency Department, Hôpitaux Pédiatriques de Nice CHU-LENVAL, Nice, France; 2 School of Medicine, Université Côte d’Azur, Nice, France; 3 EA 3279, “Santé Publique, Maladies Chroniques et Qualité de Vie”, Aix Marseille Université, Marseille, France; 4 Délégation à la Recherche Clinique et à l’Innovation, Centre Hospitalier Universitaire de Nice, Nice, France; 5 i-Virtual SAS, Metz, France

**Keywords:** eHealth, Observational Study, Paediatric intensive & critical care, Telemedicine, Triage

## Abstract

**Introduction:**

Vital signs such as heart rate (HR) and respiratory rate (RR), crucial for clinical assessment, are often challenging to measure in paediatric populations. Remote photoplethysmography (rPPG), a video-based measurement tool, has demonstrated accuracy in adults. The objective of this study is to compare HR and RR measurements obtained using rPPG with those from standard clinical monitoring in a paediatric population.

**Methods and analysis:**

This is a monocentric, prospective study enrolling 600 paediatric participants. Each participant will have standard monitoring electrodes (ECG/impedance) placed on the chest while seated facing a camera for rPPG recording. Simultaneous HR and RR measurements will be recorded over periods of 30 and 60 s using both the standard monitor and the rPPG device. The intraclass correlation coefficient will be calculated to assess agreement between the rPPG and standard monitor measurements.

**Ethics and dissemination:**

The study protocol has been approved by the French Agency for the Safety of Health Products (ANSM (Agence nationale de sécurité du médicament) registration no. IDRCB 2023-A02524-41) and by a French ethics committee (CPP Sud Méditerranée III at 29 August 2024, n°2024-A01324-43). The study’s findings will be published in peer-reviewed journals and disseminated at national and international conferences and through press releases.

**Trial registration number:**

Clinical Trials Registry (NCT06231654).

**STRENGTHS AND LIMITATIONS OF THIS STUDY**

- This study evaluates the application of a remote photoplethysmography (rPPG) imaging system in a real-world clinical setting.
- A diverse patient population, encompassing various age groups, genders and skin phototypes, is included.
- A standard acquisition system is employed as a control measure.
- A limitation of rPPG is the requirement for minimal patient movement during measurement, a challenge particularly in children.

## Introduction

 In paediatric clinical evaluation, the inherent vulnerability of children compels clinicians to assess their clinical condition quickly and efficiently. Initial measurement of vital signs allows the clinician to promptly determine whether a patient is stable. Particularly, heart rate (HR), respiratory rate (RR), oxygen saturation and blood pressure serve as objective indicators of the child’s circulatory and respiratory status.^1^ In clinical practice, manual measurements have become obsolete. Commonly used methods for measuring vital signs—such as using ECG electrodes or pulse oximetry sensors for HR and thoracic straps or chest electrodes for RR—can present practical challenges.^2^ In emergency settings, accurately measuring these vital signs requires specialised equipment, adequate staffing and sufficient time—all of which are often limited due to high patient volumes. Moreover, these measurements often occur under suboptimal conditions, such as when children are agitated or in pain. This can lead to unreliable readings precisely when accuracy is essential for assessing clinical severity. Photoplethysmography detects variations in blood volume within microvascular tissue beneath the skin’s surface. Each heartbeat induces changes in arterial and arteriolar blood volume, measurable optically.^3 4^ Conventionally, this technique uses a LED sensor placed on a finger, ear or forehead.

For adult populations, innovative methods of vital sign measurement have emerged, demonstrating acceptable reliability and accuracy compared with conventional reference techniques. Among these is remote photoplethysmography (rPPG), a method allowing rapid assessment of vital signs through a simple facial recording captured by a camera. rPPG is an innovative, non-invasive optical approach that measures pulse waves generated by cardiac activity through peripheral blood perfusion. This technology offers significant advantages: ease of use, low cost, safety, convenience and the capacity to measure multiple physiological parameters simultaneously. The company I-Virtual has significantly contributed to advancing rPPG technology, which relies on analysing variations in the facial skin’s colour spectrum to extract photoplethysmographic signals corresponding to vital signs.^5^ Their web application, Caducy, using rPPG technology, can be accessed on various devices and browsers. The operation of the rPPG method through this application is straightforward and rapid: the user places the camera (of a smartphone, tablet or computer) at an appropriate distance to capture a selfie (ensuring the entire face is visible on the screen). The application then analyses and provides vital sign parameters within 30 to 60 s.^6^ On 25 January 2023, I-Virtual’s Caducy received CE Class IIa certification as a medical device for vital sign measurement via webcam, making it the first company worldwide to obtain CE marking for this specific type of technology.^7 8^ However, the use of rPPG technology has not yet been investigated in paediatric patients. Given its ease of use, rapidity, minimal equipment needs, absence of physical contact and existing certification for adult use, rPPG (via Caducy) could potentially address the challenges of measuring vital signs in paediatric settings.

## Method

### Study aim

The aim of the study is to evaluate the feasibility and accuracy of using rPPG technology through the Caducy application in paediatric patients.

### Study design

This is a prospective, interventional, monocentric study conducted at Lenval University Children’s Hospital, Nice (France (FR)). We plan to enrol 600 paediatric patients admitted to the paediatric emergency department (PED).

#### Eligibility criteria

Eligibility and exclusion criteria are listed in table 1.

**Table 1:** List of eligibility and exclusion criteria

Eligibility criteria	Exclusion criteria
Age 3 to 17 years Admitted to the PED Signed consent by one of the two parents or the holder of parental authority Affiliation to a national social security scheme	Presence of vital distress Absence or withdrawal of parental consent Patients with neurocognitive conditions Medical history of cardiac arrhythmia, pathological tremors, muscle spasms, scleroderma Patients whose parents or legal guardians do not understand and/or speak French

PED, paediatric emergency department.

### Study procedure

**Figure 1:**
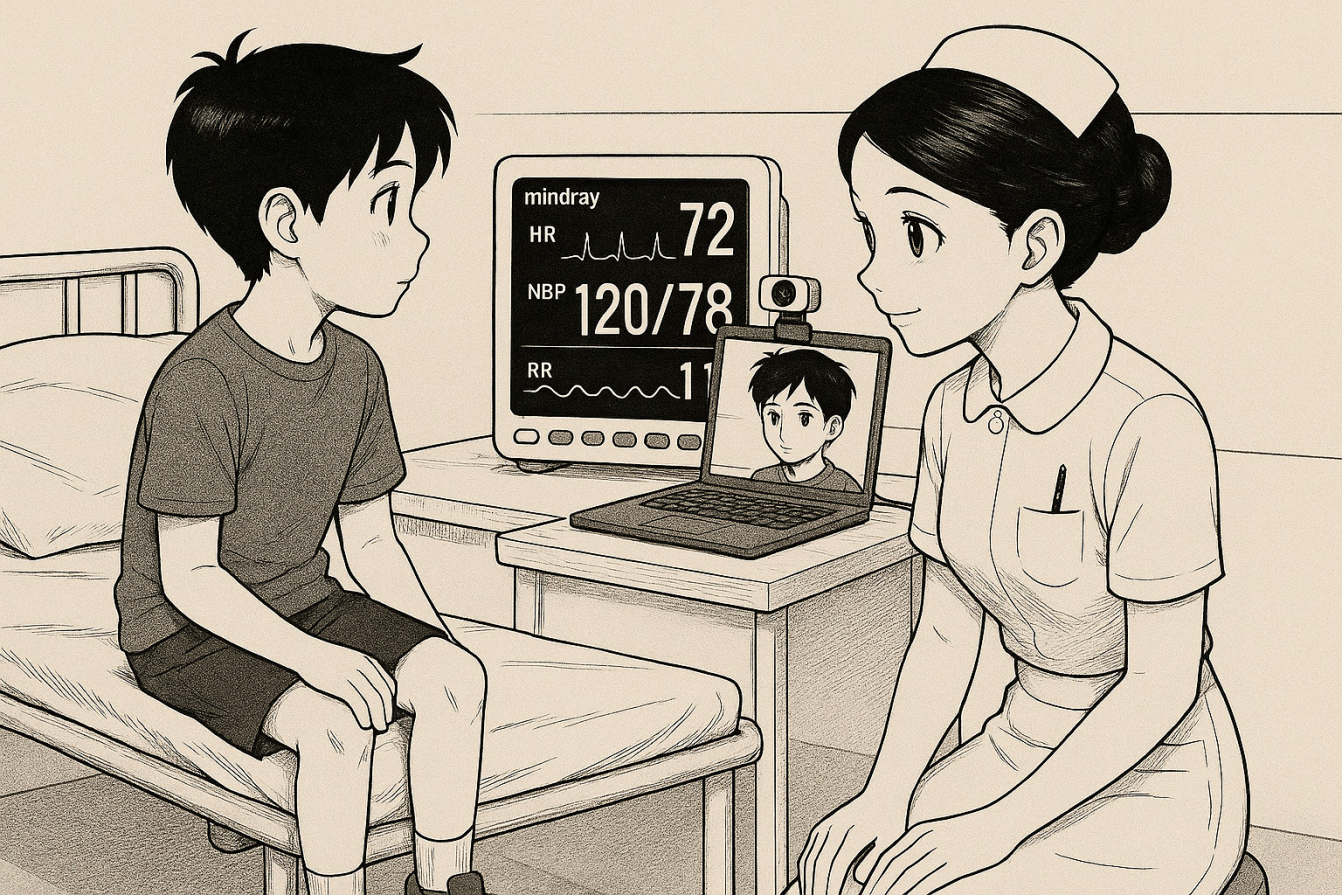
HR and RR measurements using Mindray monitor and Caducy app. The illustration used in this article was generated by an AI-based tool (OpenAI’s ChatGPT) at the explicit request of the authors. This image was created specifically for this work and is free from third-party copyright restrictions. The authors retain unrestricted rights to reproduce, distribute and display this image for academic, clinical and educational purposes. HR, heart rate; NBP, non-invasive blood pressure; RR, respiratory rate.

Patients participating in the study will be recruited from our PED. Every patient presenting to PED will be triaged by the admitting nurse. During the study period, any admission meeting the inclusion and exclusion criteria will be eligible for enrolment in our protocol during the day, depending on the availability of the clinical study technician. On arrival, an initial clinical assessment will be performed. A clinical study technician will then explain the objective and procedures of the study to the parents and to the child if they are of sufficient age and maturity to understand. After obtaining informed consent and registering the child, participants will be seated with their parents in a quiet office, where lighting conditions will be optimised to ensure accurate functioning of the Caducy application. The following data will then be collected: sex, age and phototype according to Fitzpatrick’s classification ranging from phototype 1 to 6.^9^ The child will be seated on a chair facing a camera connected to a laptop running the Caducy Medical Device software. Simultaneously and according to standard clinical procedure, three thoracic ECG electrodes will be placed on the patient, used to measure both HR and RR. The first two electrodes will be positioned on the mid-clavicular lines at the level of the second intercostal spaces, while the third electrode will be placed subcostally on the left side. The electrodes will then be connected to a Mindray ePM10 monitor. Once the child is calm and correctly positioned, the Caducy application will be initiated. Simultaneously, the Mindray monitor will record vital signs. To facilitate patient cooperation, hypnotic visual stimuli—such as kaleidoscope-type videos—will be displayed during image capture; these videos will have a dark tone to specifically reduce interference from ambient lighting. After 30 and 60 s, the HR and RR values obtained from the Caducy application (using the rPPG method) will be collected. Concurrently, HR and RR values from the Mindray monitor will also be recorded for comparison and clinical purposes (figure 1). The total duration of each patient’s participation is estimated at approximately 20 min, including preparation and data collection.

### Medical device

The reference device consists of an approved and validated Mindray ePM10 monitor.^10^

- HR will be measured using an ECG system (unit: beats per minute).
- RR will be measured using thoracic electrodes (unit: respirations per minute).

The Caducy Medical Device Software is the system used for the rPPG measurements. This software operates using an algorithm developed by I-Virtual.^11^

The measurement process consists of four successive steps:

- Step 1: Determination of the region of interest (ROI). This step involves identifying the facial skin area for HR analysis and delineating the upper chest region for RR assessment.
- Step 2: The facial ROI images are analysed to measure the red, green and blue colour channels.
- Step 3: The algorithm tracks the variations in these colour channels and the movements of the upper chest, over a defined time interval, to determine respectively the physiological parameters, HR and RR.
- Step 4: The calculated HR and RR values are consolidated into a structured digital record, each assigned a unique identifier. This record is then securely stored in a centralised health database hosting, ensuring data integrity, traceability and compliance with data protection standards.

### Primary endpoint

Analysis of reliability between HR measured by the gold standard (ECG via Mindray ePM10) and HR measured by rPPG (using the Caducy application) following a 30-s recording period. Up to two measurement attempts will be permitted for each method. If a successful measurement is not achieved within two attempts for either method, the primary endpoint will be considered unevaluable for that participant.

### Secondary endpoints

- Reliability between gold standard (ECG) HR measurements and rPPG HR measurements using a recording period of 60 s.
- Reliability between gold standard (impedance RR measure via Mindray ePM10) RR measurements and rPPG RR measurements using a recording period of 60 s.
- Reliability between gold standard RR measurements and rPPG RR measurements using a recording period of 30 s.
- Reliability of HR measurements between the Mindray ePM10 and rPPG over 30 s, stratified by age group (3 years to 7 years, 7 years to 12 years and 12 years to 18 years) and by Fitzpatrick skin phototype classification.
- Reliability of RR measurements between the Mindray ePM10 and rPPG over 30 s, stratified by age group (3 years to 7 years, 7 years to 12 years and 12 years to 18 years) and by Fitzpatrick skin phototype classification.
- Parental satisfaction with the use of the Caducy application, assessed using a 5-point Likert scale (1=‘Not at all useful’ to 5=‘Extremely useful’).
- Participant satisfaction (children/adolescents aged 3–17 years) with the use of the Caducy application, assessed using a 5-point Likert scale (1=‘Not at all useful’ to 5=‘Extremely useful’).
- Rate of measurement failure for each technique (rPPG and thoracic electrodes), defined as the inability to obtain a valid measurement value or the presence of clearly erroneous measurements attributable to poor signal quality or acquisition failure.

### Statistical analysis

Analyses will be performed by a biostatistician from the Department of Clinical Research at the CHU of Nice. The assumptions underlying each statistical test will be verified prior to analysis. Results will be considered statistically significant at the 5% level (p<0.05), unless specified otherwise. Analyses will be conducted using SAS Enterprise Guide V.7.1 (SAS Institute Inc., Cary, NC, USA). Descriptive statistics will be calculated for the study population and key study parameters. Categorical variables will be presented as absolute and relative frequencies, with 95% CIs where appropriate. Quantitative variables will be presented using means, SD, medians and IQRs. A flowchart illustrating participant flow (including numbers included and followed up) will be provided. Reliability will be assessed using the intraclass correlation coefficient (ICC) with a 95% CI to measure the agreement between the two measurement systems. Missing data will not be imputed.

### Sample size

We hypothesise an ICC of 0.90. To estimate this ICC with a precision of ±2% and considering a type I error of 5% and a type II error of 10%, the required sample size is 430 participants.^12^ To account for potential technical issues (10%), to facilitate subgroup analyses and considering our active patient pool, the total targeted sample size in this study will be 600 participants.

### Data management

To allow the collection of study-related data, a specific case report form (CRF) will be created. The design of this CRF will be developed by the clinical research associate (CRA) responsible for the study promotion and the data manager from the Clinical Research and Innovation Delegation (DRCI) at the CHU of Nice. This development will be done in collaboration with, and under the responsibility of, the principal investigator. The study-related data will be recorded in an electronic CRF (e-CRF). The e-CRF will be built by the data manager of the DRCI (using the RedCap software) based on the finalised paper CRF. The setup and implementation of the e-CRF for data collection, including user training, will be the responsibility of the DRCI. The investigators will be responsible for collecting and entering study data directly into the e-CRF, with the assistance of the study’s CRA. Quality control will be conducted on the data within the e-CRFs by CRAs from the DRCI during planned monitoring visits. The data will be secured through specific access rights based on the roles of the individuals involved in the study. Once data entry is complete, data validity and consistency checks will be performed by the data manager of the DRCI, and data queries will be issued as needed. Throughout the study, any changes made to the database will be tracked (via audit trail). At the end of the quality control process, the database will be locked (frozen) and signed by the principal investigator, the data manager and the DRCI physician. From this point onward, no further changes to the data can be made. The locked database and the data management report will then be transferred to the statistician for statistical analysis.

## Discussion

If results align with our hypothesis, this study could support using this device for HR and RR measurements, key parameters in paediatric management. This device could eventually be made available for use by parents, during teleconsultations, or by emergency services. Remote, contactless measurement of key vital signs may help address one of the main limitations of paediatric telemedicine: the lack of objective physiological data. This could, in turn, support clinical decision-making and triage in certain situations.^13 14^ However, further studies are needed to validate this device specifically in paediatrics before widespread implementation. The study poses no risk to routine clinical care, as rPPG measurements are supplemental to the standard nursing assessment. The use of rPPG consists only of a recording. The admitting nurse will conduct the standard interview and examination. Study procedures will not delay the child’s care. The only requirement for the child is to follow instructions and remain still during the brief period of vital sign collection. A maximum of two attempts will be made for each measurement method (rPPG or ECG electrodes).

## Ethics and dissemination

Ethics approval has been obtained from the French Agency for the Safety of Health Products (ANSM registration no. IDRCB 2023-A02524-41) and from the French Ethics Committee ‘CPP Sud Méditerranée III’ at 29 August 2024, n°2024-A01324-43. It will be conducted according to the European Good Clinical Practice (GCP) recommendations, the general ethical principles of the Declaration of Helsinki and specific French regulations. Participation in this study is voluntary, and each child and their parents will be informed of the purpose and content of the study and will complete a written informed consent form before being recruited for the study. We will publish the findings in peer-reviewed scientific journals and present the study findings at national and international professional conferences and through press releases.
