# Supplementary material for: Measurement of heart rate and respiratory rate using remote photoplethysmography in paediatrics: a prospective comparative trial protocol – ‘rMonitoped1’
Source: BMJ Open. 2026 Mar 19;16(3):e104840. doi: 10.1136/bmjopen-2025-104840 (PMC13007067; doi:10.1136/bmjopen-2025-104840)
Supplement: Supplementary file 1 [file bmjopen-2025-104840.reviewer_comments.pdf]

PEER REVIEW HISTORY

BMJ Open publishes all reviews undertaken for accepted manuscripts. Reviewers are asked to complete a checklist review form and are provided with free text boxes to elaborate on their assessment. These free text comments are reproduced below.

ARTICLE DETAILS

Title (Provisional)

Measurement of heart rate and respiratory rate using remote photoplethysmography in paediatrics: a prospective comparative trial protocol — “rMonitoped1”

Authors

Haas, Sébastien; Giaccardi, Hugo; Ancey, Barbara; Freyssinet, Emma; Baranton, Emma; Joulie, Aline; bendimerad, Imad; Olla, Marco; De Oliveira, fabrice; Moussaoui, Abdelhak; Boyer, Laurent; Fontas, Eric; Tran, Antoine

---

VERSION 1 - REVIEW

**Reviewer**	**1**
**Name**	**POUSSEL, Mathias**
**Affiliation**	**CHRU de Nancy, Department of Pulmonary Function Testing and Exercise Test**
**Date**	**10-Jun-2025**
**COI**	**None**

The pape is a study protocol, aimed to compare HR and RR obtained using rPPG with those frome standard clinical monitoring (Gold Standard for now) in a paediatric population .

Intro (p4/20) :

Line20

« Moreover, these measurements often occur under suboptimal conditions, such as when the child is agitated. »

I woul also precise, in addition of « agitation », painfull of children that can also contribute to suboptimal measurements in children.

Line35

« i-Virtual »

I expect this is a registred brand ? if so, please add

Method (p5/20)

Study procedure

Line 50

« During the study period, any admission meeting the inclusion criteria will be eligible for enrolment in our protocol. »

As the protocol is « based » in the PED. Will you propose the protocol 24h/24h ? because it is a paediatric population, it could be understandable that the protocole will only be proposed during the day (i.e. not the night). Please precise.

Line 60

« …phototype according to Fitzpatrick's ». For this study will you enrolled all phototype (i.e. frome 1 to 6) ? or just 1 to 4 ? Because type 5 and 6 may be more difficult to analyze and maybe need a greater number of such phototype patients. Could you

p6/20

You describe the standard clinical procedure for HR (i.e three ECG electrodes) but you don't describe the standard clinical procedure for RR. Please precise in the manuscript in the para study procedure (in addition of what you present for RR in the « Medical Device » para).

Discussion Should may be a bit longer regarding to the impact of such a study on telecs. Indeed, tomorrow, with such validated device, the number of telecs could be more important.

VERSION 1 - AUTHOR RESPONSE

Response to reviewer 1.

Reviewer 1 question 1 R1Q1/The paper is a study protocol, aimed to compare HR and RR obtained using rPPG with those from standard clinical monitoring (Gold Standard for now) in a paediatric population.
Intro (p4/20) :
Line20
« Moreover, these measurements often occur under suboptimal conditions, such as when the child is agitated. »
I would also precise, in addition of « agitation », painful of children that can also contribute to suboptimal measurements in children.

R1Q1/A1
Thank you for this comment. We have made the following revision, "Introduction" section: "Moreover, these measurements are often performed under suboptimal conditions, such as when the child is agitated or in pain."

R1Q2/Line35
« i-Virtual »
I expect this is a registred brand ? if so, please add ®

R1Q2/A2
Thank you for this comment. We added "®" each time « i-Virtual ®» was mentioned in the text

R1Q3/Method (p5/20)
Study procedure
Line 50
« During the study period, any admission meeting the inclusion criteria will be eligible for enrolment in our protocol. »
As the protocol is « based » in the PED. Will you propose the protocol 24h/24h ? because it is a paediatric population, it could be understandable that the protocole will only be proposed during the day (i.e. not the night). Please precise.

R1Q3/A3
Thank you for this comment. We have made the following revision, "Method/Study procedure" section:
"During the study period, any admission meeting the inclusion and exclusion criteria will be eligible for enrollment in our protocol during the day, depending on the availability of the clinical study technician."

R1Q4/Line 60
« …phototype according to Fitzpatrick's ». For this study will you enrolled all phototype (i.e. frome 1 to 6) ? or just 1 to 4 ? Because type 5 and 6 may be more difficult to analyze and maybe need a greater number of such phototype patients. Could you

R1Q4/A4
Thank you for your question. Indeed, the adult data did not demonstrate sufficient accuracy within this subgroup due to limited available data. However, we chose to include all skin phototypes and to stratify the analyses according to the Fitzpatrick classification. We added precision in the text as follows, "Method/Study procedure" section:
"The following data will then be collected: sex, age, and phototype according to Fitzpatrick's classification ranging from phototype 1 to 6."

As specified in one of the secondary endpoints, we plan to calculate **"**Concordance of HR and RR measurements between the Mindray® ePM10 and rPPG over 30 seconds, stratified by age group (3-6 years, 7-12 years, 13-17 years) and by Fitzpatrick skin phototype classification."

As indicated in the text "Method/Sample size" section, we calculated our sample size based on an assumption of an intraclass coefficient of 0.90. We did not adjust this calculation for the potentially limited number of patients with skin types 5 & 6, whose frequency within our cohort is unknown

R1Q5/ p6/20
You describe the standard clinical procedure for HR (i.e three ECG electrodes) but you don't describe the standard clinical procedure for RR. Please precise in the manuscript in the para study procedure (in addition of what you present for RR in the « Medical Device » para).

R1Q5/A5

Thank you for this comment. In fact, the same electrodes are used to measure both HR and RR. We have therefore made the corresponding modification in the "Method/Study Procedures" section:
"The child will be seated on a chair facing a camera connected to a laptop running the Caducy® Medical Device software. Simultaneously, and according to standard clinical procedure, three thoracic ECG electrodes will be placed on the patient, used to measure both HR and RR."

R1Q6/Discussion Should may be a bit longer regarding to the impact of such a study on telecs. Indeed, tomorrow, with such validated device, the number of telecs could be more important.

R1Q6/A6
Thank you for this comment. We have made the following revision:
"This device could eventually be made available for use by parents, during teleconsultations, or by emergency services. Remote, contactless measurement of key vital signs may help address one of the main limitations of paediatric telemedicine: the lack of objective physiological data. This could, in turn, support clinical decision-making and triage in certain situations."

Thank you very much for your time and your expertise.
